# Extracellular status of thrombospondin-2 in type 2 diabetes mellitus and utility as a biomarker in the determination of early diabetic kidney disease

**DOI:** 10.1186/s12882-023-03216-z

**Published:** 2023-05-31

**Authors:** Zhenzhen Lin, Didong Zhang, Xinxin Zhang, Wanxie Guo, Wenjun Wang, Yingchao Zhang, Zhen Liu, Yanxue Bi, Maolan Wu, Zhuofeng Lin, Xuemian Lu

**Affiliations:** 1 The 3rd Affiliated Hospital of Wenzhou Medical University (Ruian People’s Hospital), Wenzhou, 325200 China; 2 School of Pharmaceutical College, Wenzhou Medical University, Wenzhou, China; 3 The 1st Affiliated Hospital of Wenzhou Medical Unversity, South Baixiang Town, Wenzhou, 325000 China; 4 Laboratory Animal Center of Wenzhou Medical University, Wenzhou, China

**Keywords:** Thrombospondin-2, Type 2 diabetes, Early diabetic kidney disease, Biomarker, Noninvasive diagnosis

## Abstract

**Objective:**

Thrombospondin-2 (TSP-2) is a multifunctional matricellular glycoprotein correlated with glucose homeostasis, insulin sensitivity, and estimated glomerular filtration rate. Investigation of the association of TSP-2 with type 2 diabetes mellitus (T2DM) and the potential diagnostic value of serum TSP-2 for detecting early diabetic kidney disease (DKD) is needed.

**Research design and methods:**

An enzyme-linked immunosorbent assay was used for detection serum TSP-2 levels in 494 Chinese T2DM subjects. The protein expression of TSP-2 in the kidney and other tissues were tested by western blotting.

**Results:**

Serum TSP-2 levels in T2DM subjects were significantly higher than in healthy individuals. Serum TSP-2 correlated positively with triglycerides, serum uric acid, creatinine, platelets, and urinary albumin-to-creatinine ratio (UACR), but negatively with estimated glomerular filtration rate, after adjusting for age, sex, and T2DM duration. Logistic regression analysis demonstrated an independent association between serum TSP-2 and early DKD. Furthermore, the high UACR identified at risk of early DKD increased significantly from 0.78 (95%CI 0.73–0.83) to 0.82 (95%CI 0.77–0.86, *p* < 0.001) when added to a clinical model consisting of TSP-2 and age. In *db/db* mice, serum TSP-2 levels were elevated. TSP-2 expression was markedly increased in the kidney tissue compared with that in *db/m* and *m/m* mice. Furthermore, serum TSP-2 expression correlated well with UACR in mice.

**Conclusions:**

TSP-2 is a novel glycoprotein associated with early DKD in patients with T2DM. The paradoxical increase of serum TSP-2 in T2DM individuals may be due to a compensatory response to chronic inflammatory and renal vascular endothelial growth, warranting further investigation.

**Supplementary Information:**

The online version contains supplementary material available at 10.1186/s12882-023-03216-z.

## Introduction

Diabetes mellitus (DM) has become a challenging global public health concern, which was considered the leading cause of end-stage renal disease and caused approximately 1.5 million deaths worldwide in 2016 [1]. Renal puncture biopsy is currently the only gold standard for staging and diagnosing diabetic kidney disease (DKD). However, its clinical applications are limited by its invasive and high cost. It is necessary to identify biomarkers for noninvasive detection of early DKD among T2DM patients, and to develop effective pharmacotherapy to prevent or delay the progression from DM to DKD.

The thrombospondin (TSP) family consists of five members (TSP-1–5) with a wide range of biological functions including angiogenesis, inflammation, osteogenesis, cell proliferation, apoptosis and transforming growth factor-beta (TGF-β) activation. Based on the oligomerization domain, it is divided into two subgroups: trimer subgroup A (TSP-1, TSP-2) and pentamer subgroup B (TSP-3, TSP-4 and TSP-5). Only data on the role of TSP-1 and TSP-2 in renal disease are available currently. These two TSPs are hardly expressed in the healthy renal cortex. However, according to animal studies, TSP-1 and TSP-2 can be up-regulated within renal diseases. In different animal models of renal disease, both TSPs have previously been shown to be essential regulators of pathophysiological changes during renal disease [2].

The study of TSP-2 in renal inflammation and angiogenesis is still controversial. On the one hand, TSP-2 acts at a distance by regulating the activity of growth factors in the cell environment, and promoting stromal angiogenesis [3]. However, it lacks TGF-β activation ability but regulates extracellular matrix remodeling and inflammation in experimental nephropathy [4]. In a mouse model of Thy1 mesangial proliferative glomerulonephritis, overexpression of TSP-2 in muscle could significantly improve inflammation by reducing glomerular TGF-β activation and formation of extracellular matrix, and inhibit the proliferation of glomerular endothelial and mesangial cells [5]. At this moment, TSP-2 is expected to be a potential inhibitor of TGF-β activation and extracellular matrix accumulation. On the other hand, some studies have confirmed that TSP-2 may have an unfavorable effect on the kidney by increasing thrombosis. Reduced accumulation of extracellular matrix and von Willebrand factor were observed in TSP-2 knockout mice, suggesting that TSP-2 may increase thrombosis by mediating the aggregation of extracellular matrix [6]. In addition, it was found that the treatment of TSP-2 did not improve the experimental chronic allograft nephropathy, but rather worsened. This effect was likely due to its anti-angiogenesis on renal microvessels. Compared with the control group, the rats treated with TSP-2 showed significantly higher glomerular and peritubular endothelial sparsity and reduced endothelial cell proliferation in the transplanted kidney, which might be related to the decreased levels of renal vascular endothelial growth factors, although TSP-2 has anti-inflammatory and TGF-β activation blocking effects [7]. In conclusion, the above results suggest that TSP-2 may be a chronic inflammatory and angiogenic protein associated with nephropathy.

Although these experimental findings are certainly of pharmacological interest relevant to urology, the physiological role of TSP-2 remains poorly understood. To explore the clinical relevance of TSP-2 in humans, we measured its serum concentrations in 494 Chinese T2DM subjects and healthy as a control, and analyzed its association with parameters of diabetes and a cluster of renal function factors. Unexpectedly, our data demonstrated a significantly increased serum level of TSP-2 in T2DM individuals and subjects with early DKD. In line with these clinical findings, we also observed an elevated TSP-2 expression in both serum and kidney tissue in rodent models of diabetes.

## Materials and methods

### Patients and clinical examinations

In is part, we followed the methods of Gan, et al. (2022). This study measured serum TSP-2 levels among 494 subjects from our previous study [8] versus 148 control cases from healthy blood donors. The diagnosis of T2DM is based on the diagnostic and classification criteria of DM proposed by the World Health Organization in 1999: Symptoms of diabetes, a blood glucose level of 11.1 mmol/L (200 mg/dL) at any time, a fasting blood glucose (FPG) level of 7.0 mmol/L (126 mg/dL) or a 2-h blood glucose level of 11.1 mmol/L (200 mg/dL) [9]. Early DKD is diagnosed according to KDOQI clinical practice guidelines using the urinary albumin to creatinine ratio (UACR) (if ≥ 30 mg/g) [10], or according to the Mogensen diagnostic and classification criteria using the urinary albumin excretion rate (UAER) in continuous urine collection (if 30–300 mg/24 h or 20–200 μg/min) was calculated of T2DM patients. All participants gave written informed consent approved by the Human Ethics Committee of Ruian People’s Hospital.

### Measurement of TSP-2 and biochemistry parameters

Serum concentrations of TSP-2 were determined using ELISA kits (Antibody and Immunoassay Service, HKU, Hong Kong). We followed the methods of Lee et al. (2021). The assay was highly specific to human TSP-2, the lowest detection limit was 0.156 ng/mL, with intra-and inter-assay coefficients of variation being < 4.6% and < 7.2%, respectively, as described elsewhere [11]. Methods of measurement at baseline including body mass index (BMI), systolic blood pressure (SBP) and diastolic blood pressure (DBP), and methods of detecting biochemistry parameters including hemoglobin A1c (HbA1c), FPG, triglyceride (TG), total cholesterol (TC), high-density lipoprotein cholesterol (HDL-c), low-density lipoprotein cholesterol (LDL-c), serum uric acid (SUA), blood urea nitrogen (BUN), serum creatinine (SCR), urinary creatinine, and urinary albumin, and the calculation formula of UACR and the estimated glomerular filtration rate (eGFR) have been described in detail elsewhere [8].

### Animal experiments

C57BL/6 J male mice were purchased from the Animal Center of Wenzhou Medical University. Sixteen-week-old male C57BLKS/J *db/db* mice (caused by a mutation in the leptin receptor), which are considered a rodent model for T2DM, were obtained from the Jackson Laboratories. In addition, age-matched wild-type C57BL/6 *db/m* and *m/m* mice (with normal glucose and lipid metabolism) were chosed as non-diabetic controls. Mice were housed in a temperature-controlled room (22 ± 1 °C) with standard food and water ad libitum in the Laboratory Animal Center of Wenzhou Medical University. All experiments were conducted under the Wenzhou Medical University guidelines for the humane treatment of experimental animals.

### Western blotting analysis

RIPA lysis buffer containing PMSF (PMSF:RIPA = 1:100) was used to extract tissue protein. Then we loaded equal amounts of protein samples on SDS-PAGE gels and transferred them onto nitrocellulose membranes. After blocking 5% milk for one hour, the membranes were washed with TBST 3 times, each for 10 min, and then incubated at 4 °C overnight with TSP-2 (1:1000) antibodies. The membranes were then incubated with HRP-conjugated secondary antibodies at 4 °C for one hour the next day. Immunoblots were visualized by enhanced chemiluminescence (Thermo Scientific) and quantified by ImageJ software (NIH).

### Statistical analysis

In this study, all statistical analyses were performed using SPSS software Version 26.0 (IBM Corporation, NY, USA). Continuous data are reported as mean ± standard deviation or median (interquartile range), and categorical data are expressed as proportions. For continuous data, one-way ANOVA or the nonparametric Kruskal–Wallis test was applied. Additionally, multiple comparisons were analysed by one-way ANOVA followed by Tukey’s HSD test, Kruskal–Wallis test followed by Dunn’s test. For categorical data, the χ^2^ test was used. Statistical analyses were performed on natural log-transformed values for all the non-normally distributed variables.

Pearson correlation coefficient for a continuous variable and Kendall tau-b correlation coefficient for categorical variable were used to analyze the correlation of TSP-2. Partial correlation analysis adjusted for age, sex, and T2DM duration was used to identify the association of TSP-2 with potential confounding factors. Variables associated with TSP-2 in the partial correlation (*p* ≤ 0.05) were entered in a multivariable logistic regression analysis to examine the independent predictive factors of early DKD. Risk factors including TSP-2, TG, SUA, SCR, eGFR, Platelets, and UACR were selected for logistic regression analysis. Model 1 is adjusted for basic factors including age, sex, T2DM duration, BMI, and drug use of statins and angiotensin converting enzyme inhibitors (ACEIs)/angiotensin receptor blockers (ARBs); Model 2 is adjusted for factors in model 1 and all parameters with significant correlation with serum TSP-2. In this study, *p* values < 0.05 were considered to indicate statistical significance, and all tests were two-sided.

## Results

### Levels of serum TSP-2 are significantly increased in T2DM patients and correlate closely with a cluster of early DKD risk factors

TSP-2 assay was used to measure serum concentrations in 494 subjects with T2DM and 148 healthy controls included in this study as shown in Table 1. Serum TSP-2 levels ranged from 0.11 to 52.12 ng/mL. Unexpectedly, T2DM subjects had significantly higher serum TSP-2 levels than healthy [7.03 (3.48–12.91) *vs.* 6.86 (5.11–8.36) ng/mL, *p* = 0.014], in addition, the levels were significantly increased in these T2DM patients with early DKD compared to those without [10.48 (7.19–15.73) *vs.* 7.03 (3.48–12.91) ng/mL, *p* < 0.001], suggesting that TSP-2 may be related to the progress of early DKD in these patients with T2DM.

**Table 1:** Baseline characteristics of the study participants with early-stage renal damage stratified UACR (if ≥ 30 mg/g)

Variables	Healthy (*n =* 148)	T2DM_DN (*n =* 154)	T2DM_No_DN (*n =* 340)	*p* T2DM_DN vs Healthy	*p* T2DM_No_DN vs Healthy	*p* T2DM_DN vs T2DM_No_DN
Male	43 (29.1%)	97 (63.0%)	262 (77.1%)	< 0.001	< 0.001	0.002
Current smoking	30 (20.5%)	77 (50.3%)	149 (43.8%)	< 0.001	< 0.001	0.204
Current drinking	38 (26.0%)	50 (32.5%)	100 (29.4%)	0.254	0.511	0.527
Physical activity	77 (52.4%)	19 (12.3%)	34 (10.0%)	< 0.001	< 0.001	0.436
Hypertension	0 (0.0%)	76 (49.4%)	123 (36.2%)	< 0.001	< 0.001	0.007
Age, years	34.30 ± 8.27	54.49 ± 9.97	50.96 ± 10.25	< 0.001	< 0.001	< 0.001
T2DM duration, months	—	102.63 ± 80.04	39.08 ± 68.80	—	—	< 0.001
BMI, kg/m^2^	21.04 ± 2.39	25.90 ± 3.22	25.23 ± 3.33	< 0.001	< 0.001	0.034
FPG, mmol/L	4.45 (4.24–4.67)	7.79 (6.64–9.85)	7.53 (6.15–9.38)	< 0.001	< 0.001	0.60
HbA1c, %	5.23 (5.04–5.43)	8.48 (7.36–10.37)	8.23 (7.20–10.40)	> 0.999	< 0.001	< 0.001
SBP, mmHg	112.87 ± 12.22	139.22 ± 19.35	130.18 ± 17.46	< 0.001	< 0.001	< 0.001
DBP, mmHg	68.92 ± 9.25	78.54 ± 10.62	75.06 ± 10.15	< 0.001	< 0.001	0.001
TG, mmol/L	0.93 (0.75–1.17)	1.57 (1.11–2.33)	1.45 (1.04–2.02)	< 0.001	< 0.001	0.364
TC, mmol/L	4.36 (4.06–4.69)	4.80 (4.10–5.45)	4.56 (3.88–5.29)	< 0.001	0.010	0.295
HDL-c, mmol/L	1.41 (1.23–1.64)	1.04 (0.89–1.18)	1.03 (0.87–1.23)	< 0.001	< 0.001	> 0.999
LDL-c, mmol/L	2.48 (2.19–2.82)	2.93 (2.31–3.42)	2.84 (2.18–3.38)	< 0.001	< 0.001	> 0.999
SUA, μmol/L	263.0 (295.0–340.3)	257.3 (328.5–386.3)	278.0 (327.0–389.0)	0.001	0.045	> 0.999
BUN, mmol/L	4.21 (4.83–5.58)	4.55 (5.40–6.32)	4.40 (5.20–6.19)	0.004	0.010	> 0.999
SCR, mg/dL	48.75 (56.0–67.0)	61.0 (68.0–77.25)	64.0 (69.0–75.0)	< 0.001	< 0.001	> 0.999
eGFR, mL/min/1.73m^2^	116.2 (123.4–132.0)	93.6 (101.6–111.4)	100.1 (106.9–114.7)	< 0.001	< 0.001	0.002
Albumin, g/L	45.80 (44.10–47.30)	41.90 (38.93–45.0)	42.30 (39.40–44.70)	< 0.001	< 0.001	> 0.999
Platelets, 10^9^/L	228.0 (200.0–260.0)	219.0 (171.3–252.0)	75.10 (40.80–177.0)	0.203	< 0.001	< 0.001
UACR, mg/g	5.28 (2.92–7.92)	61.89 (44.05–104.48)	9.92 (6.50–15.21)	< 0.001	< 0.001	< 0.001
TSP-2, ng/mL	6.86 (5.11–8.36)	10.48 (7.19–15.73)	7.03 (3.48–12.91)	< 0.001	0.014	< 0.001
Use of statin	—	46 (29.9%)	76 (22.4%)	—	—	0.091
Use of ACEIs/ARBs	—	85 (55.2%)	138 (40.6%)	—	—	0.003
Type of antidiabetic therapy						
No	—	27 (23.9%)	163 (42.8%)	—	—	< 0.001
Insulin	—	6 (5.3%)	16 (4.2%)	—	—	0.607
OHA	—	46 (40.7%)	132 (34.6%)	—	—	0.265
Insulin + OHA	—	72 (18.9%)	35 (31.0%)	—	—	0.009

Data are presented as mean ± SD, or median (interquartile range)

*BMI* Body mass index, *FPG* Fasting plasma glucose, *HbA1c* Hemoglobin A1c, *SBP* Systolic blood pressure, *DBP* Diastolic blood pressure, *TG* Triglyceride, *TC* Total cholesterol, *HDL-c* High-density lipoprotein cholesterol, *LDL-c* Low-density lipoprotein cholesterol, *SUA* Serum uric acid, *BUN* Blood urea nitrogen, *SCR* Serum creatinine, *eGFR* Estimated glomerular filtration rate, *UACR* Urinary albumin-to-creatinine ratio, *TSP-2* Thrombospondin-2, *OHA* Oral hypoglycemic agent

Consistent with the change of age, a higher prevalence of hypertension was observed in these subjects, following a longer duration of T2DM [(102.63 ± 80.04) *vs.* (39.08 ± 68.80) months ng/mL, *p* < 0.001], and a lower eGFR [100.1 (106.9–114.7) *vs*. 93.6 (101.6–111.4) mL/min/1.73m^2^, *p* = 0.002]. However, no obvious differences in FPG and Albumin, as well as blood lipid parameters including TG, TC, HDL-c and LDL-c, and renal function parameters including SUA, BUN and SCR, were observed in these T2DM subjects with or without early DKD (all *p* > 0.05).

To investigate whether TSP-2 is related to the pathogenesis of early DKD in patients with T2DM, we next investigated the relationship between serum TSP-2 levels and a cluster of anthropometric parameters and renal function parameters. Correlation analysis showed a significant positive association of serum TSP-2 levels with SBP, TG, platelet,and renal function parameters including SUA, SCR, and UACR, respectively (all *p* < 0.05), but a negative association with eGFR (*p* < 0.05), after adjusting for age. Furthermore, the positive correlation of serum TSP-2 with these parameters, except for SBP, remained significant even after adjusting for age, sex, and T2DM duration (Table 2). Although there was a significant difference in anti-diabetic treatments (Table 1), no correlation was found between the anti-diabetic treatments and serum TSP-2 levels (Table 2). These results suggested that TSP-2 is closely ralated to renal function in patients with T2DM.

**Table 2:** Correlation between serum TSP-2 (log transformed) with various parameters (*n =* 494)

	Serum TSP-2 ^a^		Serum TSP-2^a^ (age-adjusted)		Serum TSP-2^a^ (age, sex, and T2DM duration-adjusted)	
Variables	*r*	*p*	*r*	*p*	*r*	*p*
Age	0.063	0.163	—	—	—	—
Sex	-0.006	0.867	-0.009	0.845	—	—
Current smoking	0.068	0.066	0.075	0.096	0.081	0.075
Physical activity	0.039	0.285	0.044	0.335	0.041	0.364
Hypertension	0.033	0.367	0.035	0.442	0.034	0.453
No antidiabetic therapy	-0.026	0.474	-0.040	0.378	-0.029	0.524
Insulin + OHA	-0.008	0.819	-0.012	0.782	-0.024	0.602
T2DM duration	0.051	0.263	0.032	0.484	—	—
BMI	0.068	0.134	0.073	0.109	0.073	0.108
FPG^a^	0.030	0.503	0.035	0.466	0.036	0.435
HbA1c^a^	-0.023	0.619	-0.006	0.891	-0.006	0.892
SBP	0.104	0.022	0.090	0.049	0.087	0.055
DBP	0.085	0.060	0.087	0.055	0.088	0.053
TG^a^	0.098	0.030	0.109	0.016	0.109	0.016
TC^a^	0.026	0.566	0.036	0.425	0.039	0.393
HDL-c^a^	-0.031	0.491	-0.044	0.338	-0.043	0.343
LDL-c^a^	-0.017	0.710	-0.007	0.874	-0.005	0.916
SUA^a^	0.135	0.003	0.144	0.002	0.145	0.001
BUN^a^	0.024	0.593	0.006	0.893	0.005	0.912
SCR^a^	0.104	0.021	0.101	0.025	0.113	0.013
eGFR^a^	-0.127	0.005	-0.111	0.014	-0.112	0.014
Albumin^a^	-0.058	0.203	-0.044	0.335	-0.048	0.294
Platelets^a^	0.098	0.030	0.095	0.037	0.095	0.036
UACR^a^	0.339	< 0.001	0.334	< 0.001	0.344	< 0.001

*OHA* Oral hypoglycemic agent, *BMI* Body mass index, *FPG* Fasting plasma glucose, *HbA1c* Hemoglobin A1c, *SBP* Systolic blood pressure, *DBP* Diastolic blood pressure, *TG* Triglyceride, *TC* Total cholesterol, *HDL-c* High-density lipoprotein cholesterol, *LDL-c* Low-density lipoprotein cholesterol, *SUA* Serum uric acid, *BUN* Blood urea nitrogen, *SCR* Serum creatinine, *eGFR* Estimated glomerular filtration rate, *UACR* Urinary albumin-to-creatinine ratio

^a^Log transformed

### Serum TSP-2 is independently associated with early DKD

To further evaluate the potential clinical values of TSP-2 for early-stage renal damage in patients with T2DM, multivariable logistic regressions with different models on the basis of variant risk factors were performed. The results of the logistic regression analysis for predicting early DKD were shown in Table 3. Logistic regression analysis included age, sex, T2DM duration, BMI, drug use of statins, ACEIs/ARBs, and TSP-2 levels at baseline. We found that the serum TSP-2 level was independently associated with development of early DKD [OR 2.26 (95% CI 1.63–3.14), *p* < 0.001], together with age [OR 1.06 (95% CI 1.03–1.09), *p* < 0.001], T2DM duration [OR 1.00 (95% CI 1.00–1.01), *p* = 0.008], and baseline BMI [OR 1.09 (95% CI 1.02–1.17), *p* = 0.015]. Results were similar after adjustment for baseline plus TG, SUA, SCR, eGFR, Platelet, and UACR levels. Serum TSP-2 level remained independently associated with early DKD [OR 1.94 (95% CI 1.24–3.04), *p* = 0.004], together with platelets [OR 1.66 (95% CI 1.06–2.60), *p* = 0.026], and UACR, a recently commonly used clinical laboratory marker [OR 2.52 (95% CI 1.82–3.49), *p* < 0.001].

**Table 3:** Multivariable logistic regression analysis showing the association of serum TSP-2 level with DN (*n =* 494)

Variables	OR (95%CI)	*p* value
**Model 1**		
Age	1.06 (1.03–1.09)	< 0.001
T2DM duration	1.00 (1.00–1.01)	0.008
BMI, kg/m^2^	1.09 (1.02–1.17)	0.015
TSP-2^a^, ng/mL	2.26 (1.63–3.14)	< 0.001
**Model 2**		
Platelets^a^, 10^9^/L	1.66 (1.06–2.60)	0.026
UACR^a^, mg/g	2.52 (1.82–3.49)	< 0.001
TSP-2^a^, ng/mL	1.94 (1.24–3.04)	0.004

^a^Log transformed. Model 1, variables included in the analysis were basic factors including age, sex, T2DM duration, and BMI, and drug use of statin, ACEIs/ARBs, and TSP-2 levels. Model 2, variables included in the analysis were those in model 1 plus TG, SUA, SCR, eGFR, Platelets, and UACR levels

**Fig. 1:**
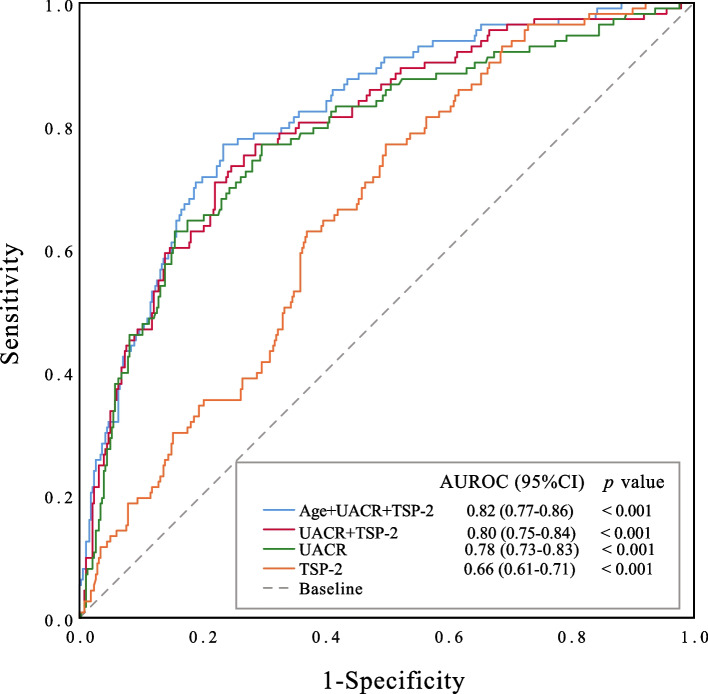
Serum TSP-2 levels and other clinical risk factors for the identification of DN in study participants with T2DM (*n =* 494)

For the lack of sensitivity and specificity in UACR (commonly used for the diagnosis of DKD), we next analysed the area under the receiving operator curve (AUROC) of both TSP-2 and UACR. Data from receiving operator characteristics (ROC) analysis indicated that the absolute value of AUROC for serum TSP-2 was 0.66 (95% CI 0.61–0.71), and for the UACR was 0.78 (95% CI 0.73–0.83), these results remained stable with a combined analysis for both TSP-2 and UACR, which reached to 0.80 (95% CI 0.75–0.84), furthermore, a larger AUROC for both TSP-2 and UACR after the addition of age at baseline was produced (Fig. 1). Taken together, serum TSP-2 levels may be a good predictor for the incidence of early DKD in patients with T2DM, and measurement of serum TSP-2 levels may be beneficial for identifying the high and low risk of DKD in patients who have undergone T2DM.

### TSP-2 expression is increased in kidney tissues of *db/db* mice and positively associated with UACR

**Fig. 2:**
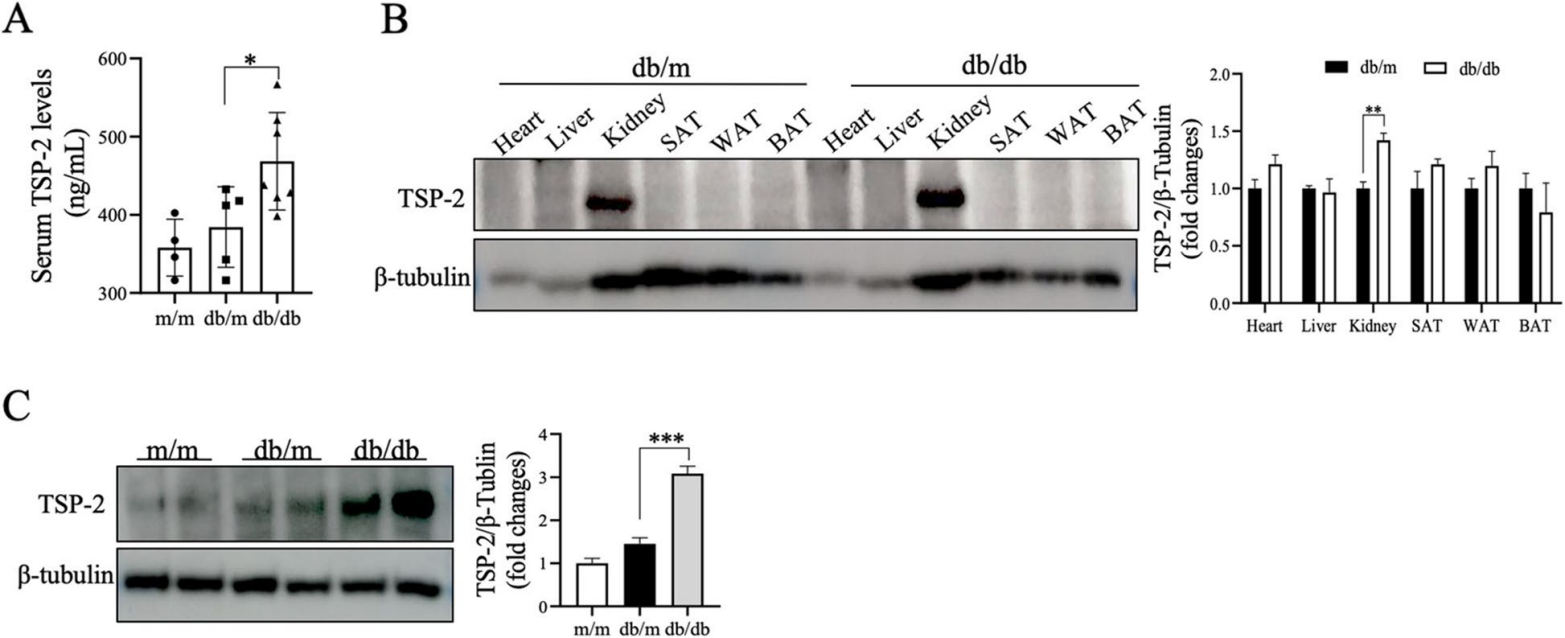
TSP-2 is expressed in kidney tissue and is elevated in *db/db* mice. **A**: Serum level of TSP-2 in different mice. **B**: Detection and comparison of TSP-2 protein expression in various tissue between *db/db* mice and their lean littermates. **C**: Comparison of TSP-2 protein expression in the kidney of m/m, db/m, and db/db mice. ^**^ *p* < 0.01, ^*^ *p* < 0.05. SAT, subcutaneous adipose tissue; WAT, white adipose tissue; BAT, brown adipose tissue

**Fig. 3:**
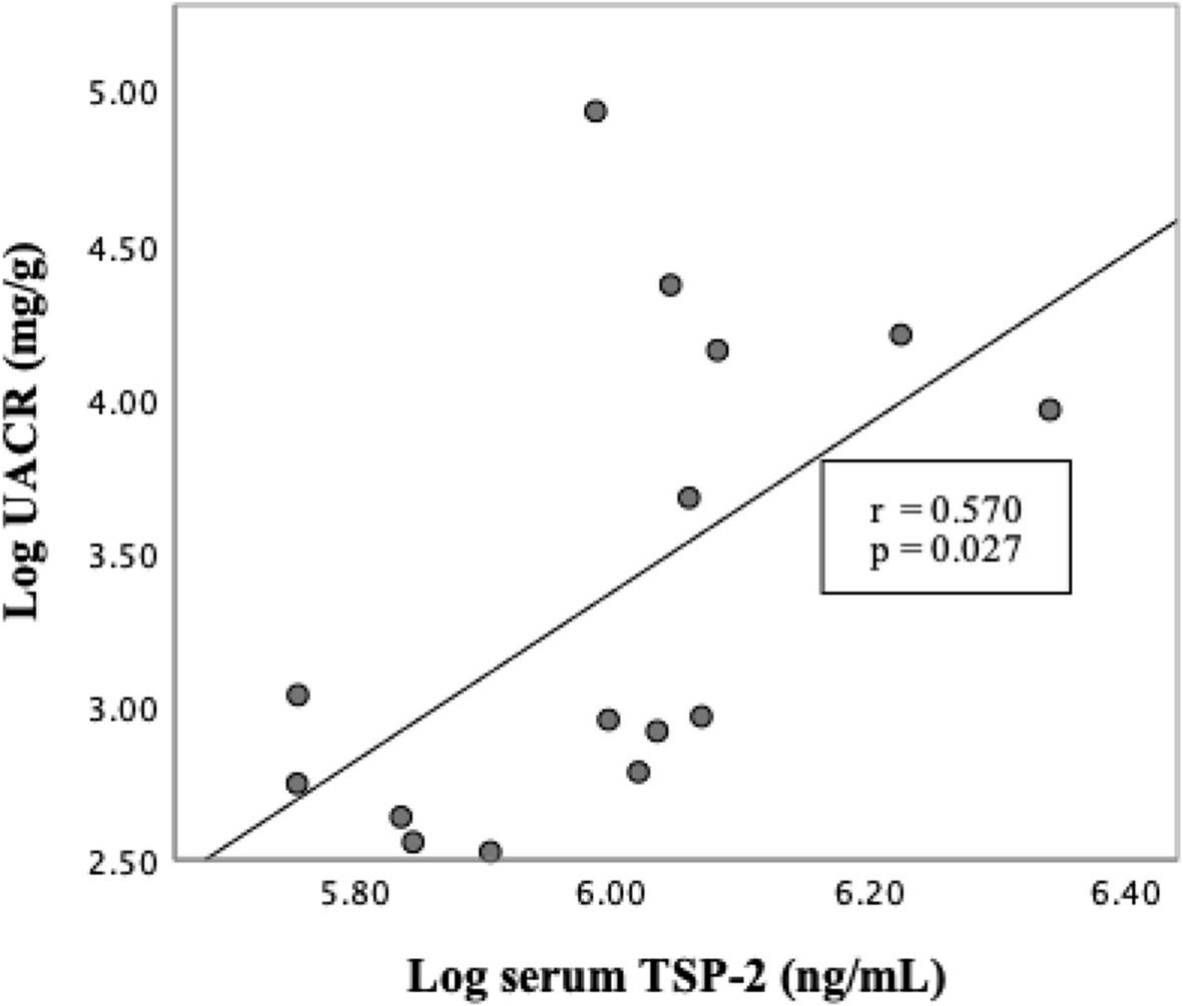
Correlation between serum levels of TSP-2 and its UACR (log transformed) in mice

Serum levels of TSP-2 were significantly increased in *db/db* mice (*p* < 0.05), (Fig. 2A). The *db/db* mice had significantly higher serum TSP-2 levels than the *db/m* mice [(468.56 ± 62.34) *vs.* (384.27 ± 51.44) ng/mL, *p* = 0.048], and *m/m* mice [(468.56 ± 62.34) *vs.* (357.88 ± 36.36) ng/mL, *p* = 0.012]. In addition, we also detected the levels of FPG, SCR, and UACR, consistent with the development of diabetes, higher FPG level, but lower levels of SCR were observed in the *db/db* mice. Similarly, the level of UACR was markedly elevated in *db/db* mice compared with *db/m* mice [(73.43 ± 34.66) *vs.* (17.78 ± 2.69) mg/g, *p* = 0.004] (Table 4). In addition, correlation analysis revealed a significant positive correlation between UACR and its serum protein concentration (Fig. 3). These data are in agreement with our clinical observations showing increased serum levels of TSP-2 in T2DM individuals.

**Table 4:** Baseline characteristics of mice

Variables	*m/m* (*n =* 7)	*db/m* (*n =* 5)	*db/db* (*n =* 7)	*db/db* vs *m/m* U (*p*)	*db/db* vs *db/m* U (*p*)	*db/m* vs *m/m* U (*p*)
FPG, mmol/L	6.11 ± 0.60	6.24 ± 0.53	31.45 ± 1.24	49.0 (0.001)	35.0 (0.003)	21.0 (0.639)
SCR, umol/L	16.00 ± 2.45	17.40 ± 2.51	9.43 ± 1.13	0.5 (0.001)	0.0 (0.003)	19.0 (0.537)
UACR, mg/g	15.05 ± 3.09	17.78 ± 2.69	73.43 ± 34.66	24.0 (0.010)	30.0 (0.004)	16.0 (0.190)
TSP-2, ng/mL	357.88 ± 36.36	384.27 ± 51.44	468.56 ± 62.34	27 (0.012)	30.0 (0.048)	14.0 (0.413)

*FPG* Fasting plasma glucose, *SCR* Serum creatinine, *UACR* Urinary albumin-to-creatinine ratio, *TSP-2* Thrombospondin-2

To further observe the physiological relevance of TSP-2 above in clinical findings, we next compared TSP-2 expression between various tissues and renal tissue in different 16-week-old male mice by Western blotting. Interestingly, TSP-2 expression was increased only in renal tissues of both *db/m* and *db/db* mice (Figs. 2B). The observation that TSP-2 was expressed in the renal tissue of *db/m* led us to examine whether TSP-2 expression was altered in diabetes. To this end, we compared the TSP-2 expression in renal tissues between 16-week-old male *db/db* mice and *db/m* mice. Notably, TSP-2 expression in renal tissue was markedly elevated in diabetic mice compared with *db/m* mice (Fig. 2C). These data suggest that renal tissue contributed, at least in part, to the elevated circulating TSP-2 levels observed in diabetic mice.

## Discussion

As a chronic, lifelong disease, T2DM is rapidly increasing in China [12], which has caused a heavy psychological and economic burden on society and individuals. Hyperglycemia, a hallmark of T2DM, could cause chronic damage and dysfunction of various tissues, especially eyes, kidneys, heart, blood vessels, and nerves. T2DM is a major factor contributing to cardiovascular disease (CVD), increasing all-cause mortality [13]. At the same time, T2DM accounts for most cases of DKD, inadequate glycemic control and ultrafiltration are two essential factors in the development of DKD [14]. The prevalence of DKD in mainland China ranged from 29.6 to 49.6% according to regional cross-sectional studies [15, 16]. And the overall prevalence of DKD in the United States from 1998 to 2014 was 17 to 35% [17]. Since DKD presents an important role to promote and increase the morbidity of CVD, early diagnosis and treatment are essential.

At present, UACR and eGFR are commonly used for the clinical diagnosis and staging of DKD. However, these two indicators lack sensitivity and specificity. UACR is susceptible to infection, fever, exercise, excessive protein intake, hypertension, hyperglycemia, heart failure, urinary tract infection, menstruation, and other factors. In addition, some patients may have decreased renal function, but the urinary albumin is still normal [18]. The eGFR cannot be measured directly and is generally estimated by the formula including SCR, age, and gender. However, the test results of SCR are also susceptible to many internal and external factors, such as metabolic level, protein intake, muscle, exercise, etc. In addition, the kidney has powerful reserve and compensation ability, and the level of SCR may be still within the normal reference range in the early stage of DKD [19]. Therefore, it is crucial to find noninvasive biomarkers with high sensitivity and specificity for the early diagnosis of DKD.

The association between TSP-2 and cardiovascular risk has recently been particularly interesting [20]. TSP-2 is a multifunctional matricellular glycoprotein characterized by anti-inflammatory, anti-angiogenic, and antiproliferative effects [4, 21–24]. However, the effect of TSP-2 in renal disease is controversial. Despite the TGF-β activation, blocking and anti-inflammatory effects presented by TSP-2, gene therapy of TSP-2 did not improve, but worsened the experimental chronic allopathy, most likely through its antiangiogenic properties on renal microvessels [7]. In contrast, a potential therapeutic effect of TSP-2 on inhibiting glomerular proliferation and inflammation, as well as TGF-β activation and extracellular matrix accumulation, has been observed in experimental mesangial proliferative glomerulonephritis [5]. Animal-based studies have demonstrated that TSP-2 was involved in the pathogenesis of renal damage in relevant mouse models [25, 26]. In accordance with the data in mice, mounting data from clinical-based studies suggest TSP-2 as a potent metabolic regulator with multiple beneficial effects on metabolic complications. Increased plasma TSP-2 has been observed in obese patients [27, 28], which may contribute to the development of obesity-related metabolic complications and nephropathy of T2DM [3, 25]. Nevertheless, the limitation is that we did not distinctly segregate anthropometric, physiological and biochemical parameters with reference to insulin resistance. Further studies are needed to elucidate the impact of TSP-2 on the association of obesity-related metabolic complications. On the other hand, this is a cross-sectional study. The findings of this current study needs to be confirmed by prospective studies or cohort studies if indicated.

In this study, we found that TSP-2 is significantly increased in T2DM patients with early DKD compared to those without early DKD, and serum TSP-2 positively correlated with renal function parameters including SUA, SCR, and UACR, but negatively with eGFR. Furthermore, TSP-2 was independently associated with early DKD by regression analysis. Besides, the predictive validity for early DKD of TSP-2 and UACR together achieved a more stable value. TSP-2 will be promising for use to make up for the deficiency of UACR. These findings indicate that TSP-2 may be a novel biomarker for early DKD in patients with T2DM. Therefore, measurement of serum TSP-2 may be beneficial for predicting early renal damage in patients with T2DM in routine clinical practice.

## Supplementary Information

**Additional file 1.**

## Data Availability

The original data may be obtained from the Lead Contact Dr. Zhuofeng Lin (the First Affiliated Hospital of Wenzhou Medical University; Email: zhuofenglin@wmu.edu.cn).

## Abbreviations

ACEIs: Angiotensin converting enzyme inhibitors
ARBs: Angiotensin-receptor blockers
AUROC: Area under the receiving operator curve
BMI: Body mass index
BUN: Blood urea nitrogen
CVD: Cardiovascular disease
DBP: Diastolic blood pressure
DM: Diabetes mellitus
DKD: Diabetic kidney disease
eGFR: Estimated glomerular filtration rate
FPG: Fasting blood glucose
HbA1c: Hemoglobin A1c
HDL-c: High-density lipoprotein cholesterol
LDL-c: Low-density lipoprotein cholesterol
OHA: Oral hypoglycemic agent
ROC: Receiving operator characteristics
SBP: Systolic blood pressure
SCR: Serum creatinine
SUA: Serum uric acid
TC: Total cholesterol
T2DM: Type 2 diabetes mellitus
TG: Triglyceride
TGF-β: Transforming growth factor-beta
TSP-2: Thrombospondin-2
UACR: Urinary albumin-to-creatinine ratio
UAER: Urinary albumin excretion rate

## References

[1] GBD 2016 Causes of Death Collaborators (2017). Global, regional, and national age-sex specific mortality for 264 causes of death, 1980–2016: a systematic analysis for the Global Burden of Disease Study 2016. Lancet..

[2] Ponticelli C, Anders HJ (2017). Thrombospondin immune regulation and the kidney. Nephrol Dial Transplant.

[3] Bornstein P, Armstrong LC, Hankenson KD, Kyriakides TR, Yang Z (2000). Thrombospondin 2, a matricellular protein with diverse functions. Matrix Biol.

[4] Hugo C, Daniel C (2009). Thrombospondin in Renal Disease. Nephron Exp Nephrol.

[5] Daniel C, Wagner A, Hohenstein B, Hugo C (2009). Thrombospondin-2 therapy ameliorates experimental glomerulonephritis via inhibition of cell proliferation, inflammation, and TGF-beta activation. Am J Physiol Renal Physiol.

[6] Kristofik N, Calabro NE, Tian W, Meng A, MacLauchlan S, Wang Y (2016). Impaired von Willebrand factor adhesion and platelet response in thrombospondin-2 knockout mice. Blood.

[7] Daniel C, Vogelbacher R, Stief A, Grigo C, Hugo C (2013). Long-term gene therapy with thrombospondin 2 inhibits TGF-β activation, inflammation and angiogenesis in chronic allograft nephropathy. PloS one..

[8] Gan J, Zheng Y, Yu QL, Zhang YC, Xie W, Shi YR (2022). Serum Lipocalin-2. Levels Are Increased and Independently Associated With Early-Stage Renal Damage and Carotid Atherosclerotic Plaque in Patients With T2DM. Front Endocrinol..

[9] Grimaldi A, Heurtier A (1999). Diagnostic criteria for type 2 diabetes. Rev Prat.

[10] KDOQI Clinical Practice GuidelinesAnd Clinical Practice Recommendations for Diabetes and Chronic Kidney Disease. Am J Kidney Dis. 2007;49:S12–154. 10.1053/j.ajkd.2006.12.005 10.1053/j.ajkd.2006.12.005 17276798

[11] Lee CH, Seto WK, Lui DT, Fong CH, Wan HY, Cheung CY (2021). Circulating. Thrombospondin-2 as a Novel Fibrosis Biomarker of Nonalcoholic Fatty Liver Disease in Type 2 Diabetes. Diabetes Care..

[12] Li Y, Teng D, Shi X, Qin G, Qin Y, Quan H (2020). Prevalence of diabetes recorded in mainland China using 2018 diagnostic criteria from the American Diabetes Association: national cross sectional study. BMJ..

[13] Rao Kondapally Seshasai S, Kaptoge S, Thompson A, Di Angelantonio E, Gao P, Sarwar N (2011). Diabetes mellitus, fasting glucose, and risk of cause-specific death. N Engl J Med.

[14] Xiong YB, Zhou LL. The Signaling of Cellular Senescence in Diabetic Nephropathy. Oxid Med Cell Longev. 2019;7495629. 10.1155/2019/7495629 10.1155/2019/7495629 PMC6794967 31687085

[15] Jia W, Gao X, Pang C, Hou X, Bao Y, Liu W (2009). Prevalence and risk factors of albuminuria and chronic kidney disease in Chinese population with type 2 diabetes and impaired glucose regulation: Shanghai diabetic complications study (SHDCS). Nephrol Dial Transplant.

[16] Lou QL, Ouyang XJ, Gu LB, Mo YZ, Ma R, Nan J (2012). Chronic Kidney Disease and Associated Cardiovascular Risk Factors in Chinese with Type 2 Diabetes. Acta Universitatis Medicinalis Nanjing.

[17] Lu B, Yang Z, Wang M, Yang Z, Gong W, Yang Y (2007). High prevalence of albuminuria in population-based patients diagnosed with type 2 diabetes in the Shanghai downtown. Diabetes Res Clin Pract.

[18] Perkins BA, Ficociello LH, Roshan B, Warram JH, Krolewski AS (2010). In patients with type 1. diabetes and new-onset microalbuminuria the development of advanced chronic kidney disease may not require progression to proteinuria. Kidney Int..

[19] Krolewski Andrzej S (2015). Progressive renal decline: the new paradigm of diabetic nephropathy. in type 1 diabetes. Diabetes Care..

[20] Morikawa N, Adachi H, Enomoto M (2019). Thrombospondin-2 as a Potential Risk Factor in a General Population. Int Heart J.

[21] Mo T, Fu Q, Hu X, Fu Y, Li J (2021). MicroRNA 1228 Mediates the Viability of High Glucose-Cultured Renal Tubule Cells through Targeting Thrombospondin 2 and PI3K/AKT Signaling Pathway. Kidney Blood Press Res.

[22] Sepahi S, Soheili ZS, Tavakkol-Afshari J, Mehri S, Hosseini SM, Mohajeri SA (2021). Retinoprotective Effects of Crocin and Crocetin via Anti-angiogenic Mechanism in High Glucose-Induced Human Retinal Pigment Epithelium Cells. Curr Mol Pharmacol.

[23] Bae ON, Wang JM, Baek SH, Wang Q, Yuan H, Chen AF (2013). Oxidative Stress-Mediated Thrombospondin-2 Upregulation Impairs Bone Marrow-Derived Angiogenic Cell Function in Diabetes Mellitus. Arterioscler Thromb Vasc Biol.

[24] Abu El-Asrar AM, Nawaz MI, Ola MS, De Hertogh G, Opdenakker G, Geboes K (2013). Expression of thrombospondin-2 as a marker in proliferative diabetic retinopathy. Acta Ophthalmol.

[25] Yeh SH, Chang WC, Chuang H, Huang HC, Liu RT, Yang KD (2015). Differentiation of type 2 diabetes mellitus with different complications by proteomic analysis of plasma low abundance proteins. J Diabetes Metab Disord.

[26] Mertens C, Kuchler L, Sola A, Guiteras R, Grein S, Brüne B (2020). Macrophage-Derived Iron-Bound Lipocalin-2 Correlates with Renal Recovery Markers Following Sepsis-Induced Kidney Damage. Int J Mol Sci.

[27] de Siqueira LT, Wanderley MSO, da Silva RA, da Silva Andrade Pereira A, de Lima Filho JL, Ferraz ÁAB (2018). A Screening Study of Potential Carcinogen Biomarkers After Surgical Treatment of Obesity. Obes Surg..

[28] Wu X, Cheung CKY, Ye D, Chakrabarti S, Mahajan H, Yan S, et al. Serum Thrombospondin Levels Are Closely Associated with the Severity of Metabolic Syndrome and Metabolic. Associated Fatty Liver Disease. J Clin Endocrinol Metab. 2022;XX:1–11. 10.1210/clinem/dgac292 10.1210/clinem/dgac292 35532410

