# Supplementary material for: Extracellular status of thrombospondin-2 in type 2 diabetes mellitus and utility as a biomarker in the determination of early diabetic kidney disease
Source: BMC Nephrol. 2023 May 31;24:154. doi: 10.1186/s12882-023-03216-z (PMC10230672; doi:10.1186/s12882-023-03216-z)
Supplement: Supplementary file 1 — Additional file 1. [file 12882_2023_3216_MOESM1_ESM.pdf]

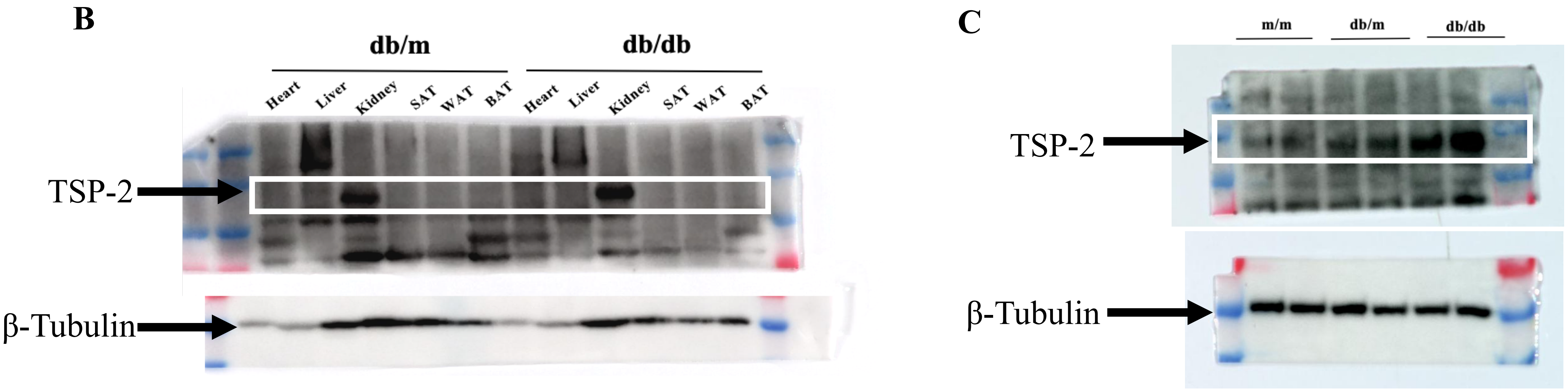

Figure 2 TSP-2 is expressed in kidney tissue and is elevated in *db/db* mice

B: Detection and comparison of TSP-2 protein expression in various tissue between *db/db* mice and their lean littermates; C: Comparison of TSP-2 protein expression in kidney of *db/db* mice.
